# Performance of Chat Generative Pre-trained Transformer-4o in the Adult Clinical Cardiology Self-Assessment Program

**DOI:** 10.1093/ehjdh/ztae077

**Published:** 2024-10-21

**Authors:** Abdulaziz Malik, Christopher Madias, Benjamin S Wessler

**Affiliations:** Cardiovascular Center, Tufts Medical Center, 800 Washington Street, Boston, MA 02111, USA; Cardiovascular Center, Tufts Medical Center, 800 Washington Street, Boston, MA 02111, USA; Cardiovascular Center, Tufts Medical Center, 800 Washington Street, Boston, MA 02111, USA

**Keywords:** Medical education, Artificial intelligence, Large language models

## Abstract

**Aims:**

This study evaluates the performance of OpenAI’s latest large language model (LLM), Chat Generative Pre-trained Transformer-4o, on the Adult Clinical Cardiology Self-Assessment Program (ACCSAP).

**Methods and results:**

Chat Generative Pre-trained Transformer-4o was tested on 639 ACCSAP questions, excluding 45 questions containing video clips, resulting in 594 questions for analysis. The questions included a mix of text-based and static image-based [electrocardiogram (ECG), angiogram, computed tomography (CT) scan, and echocardiogram] formats. The model was allowed one attempt per question. Further evaluation of image-only questions was performed on 25 questions from the database. Chat Generative Pre-trained Transformer-4o correctly answered 69.2% (411/594) of the questions. The performance was higher for text-only questions (73.9%) compared with those requiring image interpretation (55.3%, *P* < 0.001). The model performed worse on questions involving ECGs, with a correct rate of 56.5% compared with 73.3% for non-ECG questions (*P* < 0.001). Despite its capability to interpret medical images in the context of a text-based question, the model’s accuracy varied, demonstrating strengths and notable gaps in diagnostic accuracy. It lacked accuracy in reading images (ECGs, echocardiography, and angiograms) with no context.

**Conclusion:**

Chat Generative Pre-trained Transformer-4o performed moderately well on ACCSAP questions. However, the model’s performance remains inconsistent, especially in interpreting ECGs. These findings highlight the potential and current limitations of using LLMs in medical education and clinical decision-making.

## Graphical Abstract

**Graphical Abstract:**
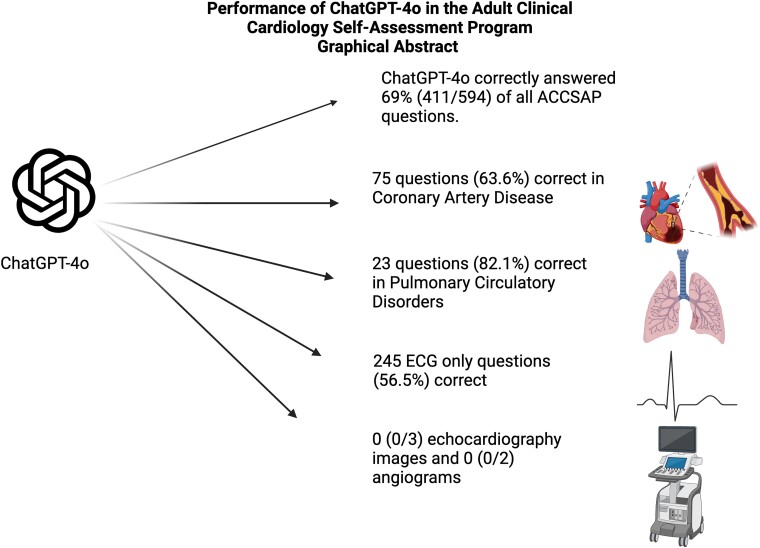

In the last 5 years, large language models (LLMs) have become widely available and increasingly sophisticated. These models have been proposed as adjunctive tools to improve medical care. Recently, Chat Generative Pre-trained Transformer 3 (ChatGPT-3) and ChatGPT-4 were tested on questions in the style of the United States Medical Licensing Exams,^1^ and while performance on general medicine questions is improving (ChatGPT-3 correct response rate 62.5 vs. 90% for ChatGPT-4), performance in subspecialty domains remains uncertain. We hypothesize that LLM performance on cardiovascular medicine questions will be lower when compared with general medicine questions because of the subspecialized complexity of the field and the need to incorporate imaging data in order to reach correct clinical decisions. With the advent of ChatGPT-4, image analysis is now possible and a new model, ChatGPT-4o (omni), released by Open AI on 13 May 2024. Chat Generative Pre-trained Transformer-4o demonstrates excellent performance across several categories such as Massive Multitask Language Understanding and Graduate-Level Google-Proof.^2^ We aimed to assess the performance of ChatGPT-4o in the Adult Clinical Cardiology Self-Assessment Program (ACCSAP), a question bank that includes imaging and is used to prepare for the general cardiology board exam administered by the American Board of Internal Medicine.

Our approach involved posing questions in the ACCSAP to ChatGPT-4o with the prompt—‘What is the answer?’ We allowed only one attempt for each question. We excluded all questions with videos, as the model cannot interpret video, but included all questions with static images. The data collection process included capturing responses to each question and identifying whether the question contained imaging data such as an electrocardiogram (ECG), angiogram still frame, computed tomography (CT) scan, or echocardiogram still frame. We further evaluated the model with a total of 25 image-only questions. These included ECGs, echocardiography images, and angiograms (20 ECGs, 3 echocardiography images, and 2 angiograms) with the prompt ‘What is the ECG/Echocardiography/Angiogram read?’. A practicing cardiologist judged the accuracy of these open-ended questions. The collected data were analysed using SPSS version 29.

Six hundred and thirty-nine questions were reviewed for inclusion. Forty-five questions (7%) were excluded because they contained video clips. Five hundred and ninety-four questions were used for this analysis. One hundred and twenty-four (20.9%) included ECG data, 6 (1.0%) included angiogram still frame data, and 10 (1.7%) included still image echocardiograms. Chat Generative Pre-trained Transformer-4o provided a conclusive answer to 594 (100%) of the questions on the first attempt and had a correct score of 411/594 (69.2%). Chat Generative Pre-trained Transformer-4o performance was noted to be lower when questions contained any imaging vs. none [83/150 (55.3%) vs. 328/444 (73.9%), *P*-value ≤ 0.001 by χ^2^ test]. Chat Generative Pre-trained Transformer-4o performed worse when ECG interpretation was required vs. not (56.5 vs. 73.3% correct, respectively, *P*-value <0.001 by χ^2^ test). When evaluating open-ended image-only, ChatGPT-4o correctly answered 4 out of 25 questions as judged by a cardiologist. It correctly read 4/20 ECGs (two normal ECGs, one ECG that showed ventricular tachycardia, and one showing polymorphic ventricular tachycardia).

Notably, ChatGPT-4o interpreted most of the ECGs and echocardiographic images in the questions; however, the answers varied in accuracy (*Figure 1*). In one question, ChatGPT-4o accurately diagnosed inferior ST-segment elevation myocardial infarction (STEMI) on the ECG and mentioned that it could lead to right ventricular failure, affecting the patient’s preload status. However, in another question, it mistook a right bundle branch block for a STEMI. Chat Generative Pre-trained Transformer-4o was able to accurately diagnose coronary vasospasm based on the clinical context and angiographic image and explained its resolution with intracoronary nitroglycerin in the next angiogram.

**Figure 1:**
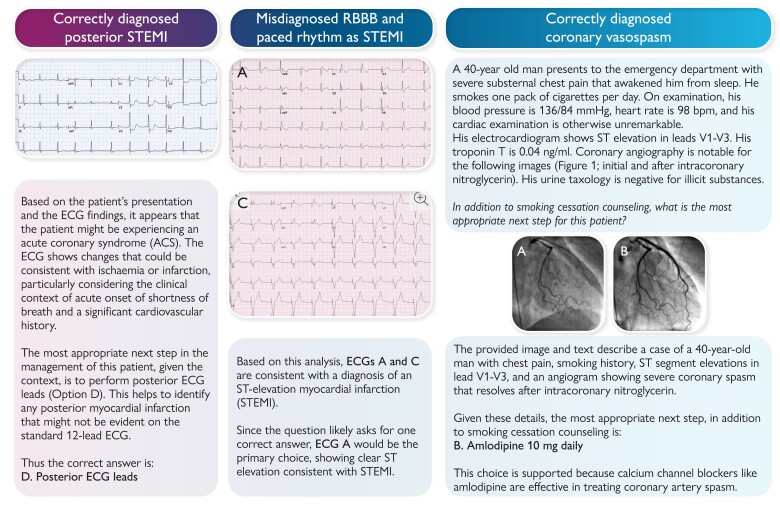
Performance of ChatGPT-4o across different cardiovascular domains in the adult clinical cardiology self-assessment program

The performance of ChatGPT-4o was variable in the different domains of the ACCSAP. It scored the best in the pulmonary section and systemic disorders. Previous research has suggested that ChatGPT scores better in general medicine than in specialized medical questions.^1^ This may explain the disparity in performance as the pulmonary section for cardiologists and systemic disorders are less specialized than heart failure and coronary artery disease (Table 1).

**Table 1:** Performance of Chat Generative Pre-trained Transformer-4o across the different domains of ACCSAP

Category	Correct answer (%)	Correct answers for Questions with ECGs (%)
Pericardial diseases	13 (72.2%)	1 (50.0%)
Congenital heart disease	22 (73.3%)	1 (50.0%)
Heart failure and cardiomyopathy	56 (65.1%)	10 (58.8%)
Miscellaneous	22 (71%)	0 out of 2 (0%)
Pulmonary circulatory disorders	23 (82.1%)	1 (50%)
Systemic disorders affecting the cardiovascular system	9 (81.8%)	2 (100%)
Arrhythmias	54 (65.9%)	32 (59.3%)
Vascular disorders	28 (71.8%)	NA
Valvular heart disease	67 (69.8%)	2 (50.0%)
Systemic hypertension and hypotension	42 (76.4%)	NA
Coronary artery disease	75 (63.6%)	21 (52.5%)

These results show evidence of expanding capabilities of the most recent LLMs and good performance on cardiology board preparation questions. As these questions are not publicly available, it is unlikely the latest models have seen them before. In a previous study assessing the performance of ChatGPT-3 with cardiology board-style questions, the LLM scored 58.8% overall.^3^ However, this prior study excluded all image-based analyses, which comprised 25% of all queries in the assessment (ChatGPT-3 cannot interpret imaging). While we did not directly compare ChatGPT-4o’s performance with ChatGPT 3, in this analysis, ChatGPT-4o correctly answered questions on the ACCSAP at a higher level when compared with a prior analysis of ChatGPT-3’s abilities on European Cardiology board-style questions.^3^

Despite improved capabilities, performance of ChatGPT-4o on cardiology-style questions remains variable. At times, ChatGPT-4o correctly identifies challenging diagnoses, while at other times the LLM misses diagnoses that seem more evident. While ChatGPT-4o responded to all the posed questions, there were significant knowledge gaps as ∼30% of these board-style questions were answered incorrectly. Chat Generative Pre-trained Transformer-4o image analysis capabilities allow the LLM to interpret medical images, including ECGs, echocardiograms, and CT scans, and verge upon how clinicians integrate imaging data into clinical decision-making. Our findings suggest that further advances in artificial intelligence augur for the continued improvement in the accuracy and medical reasoning of LLMs.

## Data Availability

The data are available upon request.
